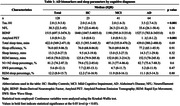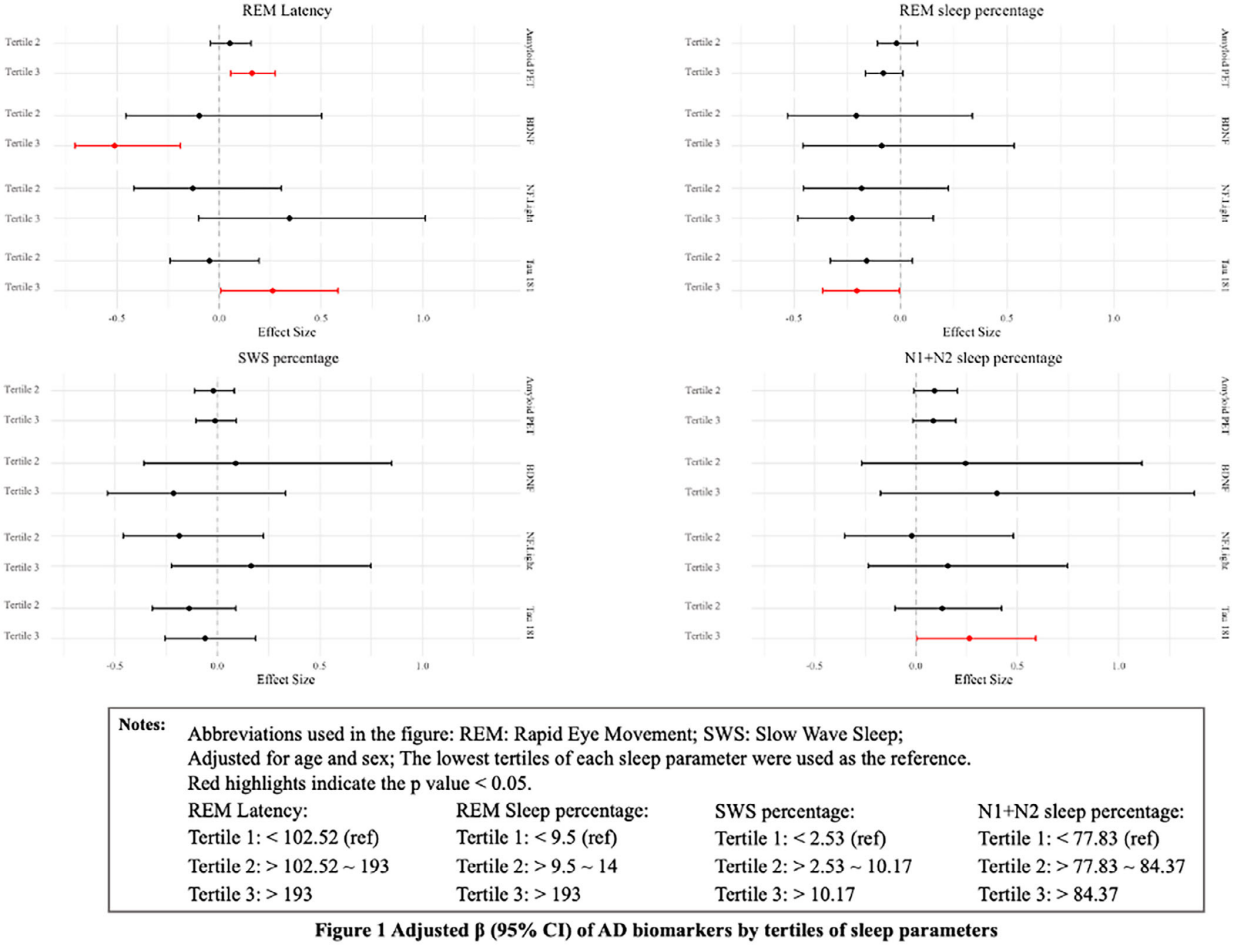# Association of Polysomnography‐Assessed Sleep Parameters with Alzheimer’s Disease Biomarkers in Older Adults

**DOI:** 10.1002/alz.088517

**Published:** 2025-01-09

**Authors:** Jiong Chen, Clémence Cavaillès, Jiangli Jin, Laura Stankeviciute, Joseph R. Winer, Song Gao, Dantao Peng, Yue Leng, Sasha Milton

**Affiliations:** ^1^ Peking University Health Science Center, Beijing City, Beijing China; ^2^ University of California, San Francisco, San Francisco, CA USA; ^3^ China‐Japan Friendship Hospital, Beijing City, Beijing China; ^4^ Barcelonaβeta Brain Research Center (BBRC), Barcelona Spain; ^5^ Stanford University School of Medicine, Stanford, CA USA; ^6^ Department of Psychiatry and Behavioral Sciences, University of California, San Francisco, San Francisco, CA USA

## Abstract

**Background:**

over the past decade, a bi‐directional relationship between sleep and Alzheimer’s disease (AD) has been increasingly recognized, with several studies suggesting a link between self‐reported sleep disturbances and AD biomarkers. However, the association between polysomnography (PSG)‐assessed sleep parameters and AD biomarkers remains unknown.

**Method:**

We examined 128 participants [mean age 70.9 (±9.7) years, 43.8% of men; 23 healthy controls (HC), 41 with mild cognitive impairment (MCI), and 64 with AD] from an outpatient memory clinic. Sleep features were derived from overnight PSG and were categorized by tertiles. Four AD biomarkers were measured, including tau‐181, Brain‐Derived Neurotrophic Factor (BDNF), and neurofilament light (NFL) obtained from blood samples, and Aβ levels measured from amyloid positron emission tomography scans. We used multivariable linear regression models to examine the associations between sleep parameters and AD biomarkers.

**Result:**

After adjustment for age, sex, APOE4 status, diabetes, smoking habits, and body mass index, participants in the highest tertile of rapid eye movement (REM) latency (>192.7 minutes) had higher tau‐181 (β = 0.25, 95% confidence interval (CI) = 0.01;0.55) and Aβ (β = 0.15, 95% CI = 0.06;0.26) levels, and lower BDNF (β = ‐0.51, 95% CI = ‐0.70;‐0.20) compared to the lowest tertile (< 98.2 minutes). Moreover, participants in the middle slow wave sleep (SWS) percentage tertile (2.5‐10.2%) had lower tau‐181 levels (β = ‐0.18, 95% CI = ‐0.34;‐0.02) compared to those in the lowest tertile (< 2.5%). We did not find any association between AD biomarkers and sleep duration, efficiency, latency, or other sleep macro‐architecture features. The association between sleep and AD biomarkers did not differ by cognitive diagnoses (i.e., NC, MCI, or AD).

**Conclusion:**

Longer REM latency was associated with higher levels of AD biomarkers in older adults with and without cognitive impairment. Future research is needed to examine the longitudinal association between sleep architecture and AD biomarkers and clarify underlying mechanisms.